# Optimizing treatment of tauroursodeoxycholic acid to improve embryonic development after *in vitro* maturation of cumulus-free oocytes in mice

**DOI:** 10.1371/journal.pone.0202962

**Published:** 2018-08-27

**Authors:** Masato Mochizuki, Kodai Miyagi, Satoshi Kishigami

**Affiliations:** 1 Faculty of Life and Environmental Sciences, University of Yamanashi, Yamanashi, Japan; 2 Advanced Biotechnology Center, University of Yamanashi, Kofu-shi, Yamanashi, Japan; China Agricultural University, CHINA

## Abstract

Cumulus-free *in vitro* maturation (IVM) provides a powerful tool to manipulate immature oocytes, but IVM oocytes lead to poor development after fertilization. Supplementation of the culture medium with tauroursodeoxycholic acid (TUDCA), a bile acid, has been reported to improve the development of embryos derived from *in vivo* fertilized (IVF) embryos after *in vitro* culture (IVC) by attenuating endoplasmic reticulum stress. However, it remains unclear if TUDCA can improve development of IVM-IVF embryos. Here, we examined whether TUDCA treatment could improve embryonic development during or after IVM. Immature GV oocytes collected from ovaries of ICR female mice that were free from cumulus cells were subjected to IVM in αMEM containing 5% FBS for 16 h. TUDCA was added to the media at varying concentrations (0–1000 μM) during IVM and IVC. TUDCA treatment during IVM reduced both MII and pronuclear (PN) rates but did not affect blastocyst rates of fertilized embryos. In contrast, TUDCA treatment during IVC significantly increased blastocyst formation rates in a concentration dependent manner. Finally, embryo transfer after TUDCA treatment revealed a significant improvement in the rates of offspring production (15% with 1000 μM TUDCA vs. 6.0% control). These results show that treatment with 1000 μM of TUDCA significantly can improve poor embryonic development of cumulus-free IVM-IVF embryos.

## Introduction

*In vitro* maturation (IVM) of mammalian oocytes provides a powerful tool for reproductive biology and assisted reproductive technologies [1]. However, the developmental potential of mature oocytes after IVM of germinal vesicle stage (GV) oocytes has been limited when compared to *in vivo* matured oocytes [2,3]. Further, although it is necessary to micromanipulate GV oocytes by removing the surrounding cumulus cells, this results in severe impairment of subsequent embryo development. Serum-containing media for IVM improves the quality of matured oocytes [4], and has been used widely [5]. Moreover, instead of serum usage, a combination of media, αMEM, and TYH can improve the quality of cumulus-free IVM oocytes in mice, which enables production of offspring from spermatocytes [6]. Alternatively, the metaphase II (MII) karyoplasts of matured oocytes must be transferred into enucleated *in vivo* matured oocytes to replace the cytoplasm [7].

Historically, bear bile was used to treat multiple diseases, including jaundice [8]. Tauroursodeoxycholic acid (TUDCA), an endogenous bile acid, relieves endoplasmic reticulum (ER) stress by acting as a chemical chaperone. TUDCA has been demonstrated to inhibit apoptosis by modulating mitochondrial membrane perturbation and/or attenuating ER stress [9,10]. Recently, it has been reported that ER stress was induced during IVM in pig, which was reduced by adding melatonin and TUDCA to improve oocyte quality and maturation rates [11]. ER stress also is induced even after *in vivo* fertilized (IVF). Treatment of embryos with TUDCA during preimplantation improves subsequent development in mice and pig by attenuating apoptosis, presumably by relieving ER stress [12–14]. Therefore, in this study, we seek to improve the efficiency of IVM-IVF technology by optimizing the timing and concentration of TUDCA to alter the rate of offspring production in IVM-IVF cumulus-free oocytes.

## Materials and methods

### Animals

Oocytes were collected from female ICR mice at 8–10 weeks of age. To generate surrogate pseudopregnant embryo transfer recipients, we mated ICR mice with vasectomized males of the same strain. ICR female mice were purchased from Japan SLC (Hamamatsu, Japan). The animals were housed under a controlled lighting condition (daily light 07:00–21:00 h) and were maintained under specific-pathogen-free conditions. On the day of the experiments or after finishing all experiments, mice were euthanized by CO_2_ inhalation or cervical dislocation and used for experiments. All animal experiments were approved by the Animal Experimentation Committee at the University of Yamanashi and were performed in accordance with the committee’s guiding principles.

### Tauroursodeoxycholic acid

Tauroursodeoxycholic acid sodium salt (TUDCA; Nacalai Tesque) was dissolved in sterile, distilled water to make a 100 mM stock solution (stored at 4°C). This stock solution was diluted into IVM and CZB culture media to make 10, 100, and 1000 μM solutions of TUDCA.

### Collection and in vitro maturation of immature oocytes

Methods for collection of GV stage oocytes and maturation were described previously [15]. Briefly, GV stage oocytes were collected from the ovaries of ICR female mice at 46 to 48 h after a 7.5 IU intraperitoneal injection of pregnant mare serum gonadotropin (PMSG; Teikokuzoki, Tokyo, Japan). Blood and fat on the removed ovaries were removed. The ovary was dissected using a 26-guage needle (Terumo Co., Tokyo, Japan), and the GV oocytes with cumulus cells were collected in 200 μl HEPES-CZB. The cumulus cells were denuded by pipetting. Small, dark, and distorted immature oocytes were removed. Denuded GV oocytes were cultured for 16 h in 50 μl of *in vitro* maturation (IVM) medium, αMEM (12571–630; Gibco), containing 5% FBS (SH30910.03; HyClone). Only oocytes that developed into MII oocytes were collected and subjected to *in vitro* fertilization (IVF). *In vivo* matured MII oocytes were collected from oviducts of ICR female mice that were superovulated due to an intraperitoneal injection of 7.5 IU PMSG followed by 7.5 IU human chorionic gonadotrophin (hCG; Teikokuzoki) 48 h later. Cumulus–oocyte complexes were placed in 200 μl of HTF medium covered with paraffin oil (Sigma-Aldrich).

### *In vitro* fertilization and subsequent embryo development

To assess the developmental potential of oocytes reaching the MII stage via IVM, the oocytes were subjected to IVF experiments. Sperm collected from the caudal epididymides of mature ICR males over 10 weeks were allowed to disperse in HTF medium and were preincubated for 1 h at 37°C under 5% CO_2_. The final sperm concentration for insemination was 1×10^6^ sperm/ml in HTF. Oocytes were fertilized *in vitro* for 6 h and were cultured in CZB for 96 h to examine their subsequent development. For IVF using *in vivo* matured oocytes, the same procedure was carried out, except that the sperm concentration was 1.0 × 10^5^ sperm/ml. Some of the IVM-IVF embryos that reached the 2-cell stage at 24 h of culture were transferred into the oviducts of day one pseudopregnant ICR recipient females that had been mated with vasectomized males.

### Statistical analysis

Development rates were compared using Z-tests, and differences between groups were considered significant when P < 0.05.

## Results

### Effect of TUDCA treatment during IVM on subsequent development

**Fig 1:**
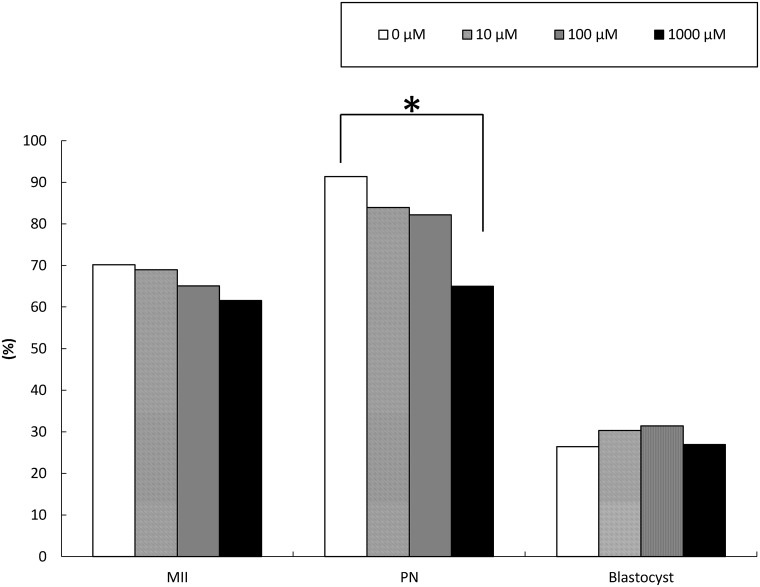
Effect of TUDCA treatment during IVM on oocyte maturation, pronuclear formation, and *in vitro* development. After IVM of GV oocytes treated with varying concentrations of TUDCA (0, 10, 100, 1000 μM), matured MII oocytes were subjected to IVF and *in vitro* culture for 96 h (N>60, respectively). Although maturation and fertilization rates decreased in a TUDCA-concentration-dependent manner, there was no difference in blastocyst formation rates or obvious abnormalities. Scale bar = 50 μm. *P < 0.05.

More than 60 cumulus-free GV oocytes were matured under 0, 10, 100 and 1000 μM TUDCA in each experimental group. Although normal MII oocytes and fertilized embryos were obtained (Fig 1), the rates of maturation and pronuclear formation (PN) decreased in the presence of 1000 μM TUDCA (Fig 1). Regardless, fertilized embryos derived from these TUDCA-treated MII oocytes developed to the blastocyst stage. Thus, these results indicate that there is no beneficial effect of TUDCA treatment on IVM-IVF of cumulus-free GV oocytes.

### TUDCA treatment increases blastocyst rates of IVM-IVF embryos

We next examined whether supplemented TUDCA shortly after IVF, which was kept up to the blastocyst stage, improved subsequent development. Treatment with 1000 μM TUDCA significantly increased blastocyst formation rate (Table 1). Interestingly, the concentrations of TUDCA required for improvement were stage-specific. The development from PN to the 2-cell stage required at least 100 μM TUDCA, but the development from the 2-cell to 4/8-cell stage required 1000 μM TUDCA, even though lower concentrations of TUDCA led to better development than control (Table 1).

**Table 1:** Optimizing TUDCA concentration based on *in vitro* development.

TUDCA (μ M)	MII	PN (%)	2 cell (%)*	4/8 cell (%)*	morula (%)*	blastocyst (%)**
0	165	124 (75)	114 (92) ^a^	76 (67) ^a^	56 (74)	42 (34)^a^
10	99	72 (73)	64 (89) ^a^	49 (76) ^a^^,^^b^	41 (84)	28 (39) ^a^^,^^b^
100	103	72 (70)	71 (99) ^b^	50 (70) ^a^^,^^b^	41 (82)	28 (39) ^a^^,^^b^
1000	102	77 (75)	76 (99) ^b^	61 (80) ^b^	50 (82)	38 (49) ^b^

* Percentages relative to the number of embryos at the former developmental stage.

** Percentages relative to the number of PN embryos

^a,b^Values differ significantly (P<0.05)

**Fig 2:**
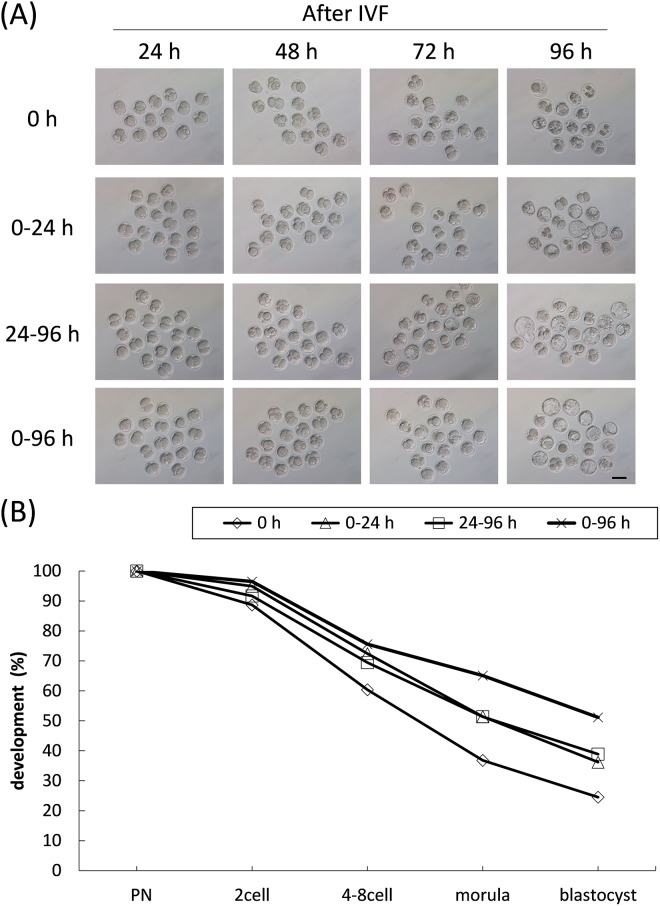
Requirements of TUDCA throughout *in vitro* culture. (A) After IVM and IVF, fertilized oocytes were subjected to *in vitro* culture for up to 96 h. During embryo culture, 1000 μM TUDCA was added at specific time periods: 0–24 h, 24–96 h, 0–96 h (N>70, respectively). Even the first treatment of TUDCA 24 h after IVF improved subsequent development. The developmental rates of each experimental group were quantified (B). Scale bar = 50 μm.

TUDCA improves the rate of two-cell embryo development to blastocysts by attenuating ER stress.^12^ Therefore, we analyzed the time required for TUDCA to improve embryonic development using 1000 μM TUDCA. Fertilized embryos were cultured without TUDCA 24 h after IVF and those embryos at the 2-cell stage were treated with TUDCA for the next 72 h, which we designated as “24–96 h” in Fig 2. Although treatment for 24–96 h led improvements relative to controls, treatment for 0-96h maximized blastocyst formation rate. These results suggest that first 24 h following IVF is also critical for TUDCA treatment as well as the following 24–96 h.

### TUDCA treatment increases offspring rates of IVM-IVF embryos

**Fig 3:**
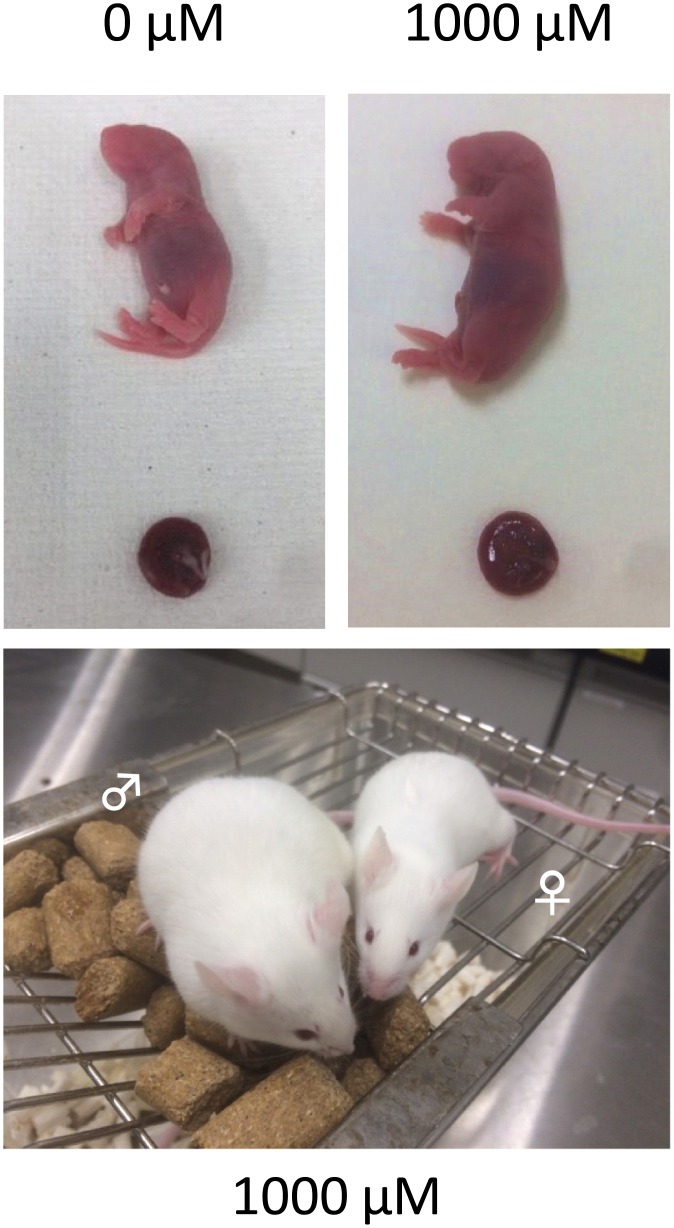
Full-term development of embryos treated with TUDCA. After IVM and IVF, fertilized embryos were subjected to *in vitro* culture with 0, 10, 100, and 1000 μM TUDCA. At the 2-cell stage, they were transferred into the oviduct of a pseudopregnant female. The offspring rates increased in a dose-dependent manner (Table 2). The pups appeared and grew normally.

To elucidate whether TUDCA treatment 24 h after IVF supported full-time development, 2-cell stage embryos treated with TUDCA were transferred into oviducts of pseudopregnant females. Consistently, offspring rates increased in a dose-dependent manner (Table 2). After birth, there were no obvious abnormalities in mice derived from TUDCA-treated embryos (Fig 3).

**Table 2:** Full-term development of IVM embryos after TUDCA treatment.

TUDCA (μM)	MII	PN (%)	2 cell (%)*	No. of ET	No. of offspring (%)**
0	220	142 (65)	126 (89)	117	7 (6.0)^a^
10	218	134 (61)	127 (95)	119	9 (7.6) ^a^^,^^b^
100	45	37 (82)	37 (100)	32	3 (9.4) ^a^^,^^b^
1000	91	45 (49)	43 (96)	34	5 (14.7)^b^

* Percentages relative to the number of PN oocytes

** Percentages relative to the number of ET embryos

^a,b^Values differ significantly (P<0.1)

## Discussion

Here we examined whether TUDCA treatment could improve development of cumulus-free IVM-IVF embryos. As is true for any chemical treatment of embryos, the timing and concentration of the chemical applied is important and should be optimized to maximize results [16]. Our results show that treatment of embryos with 1000 μM TUDCA significantly increased both blastocyst formation and offspring production rates when it was applied during preimplantation development following IVF, but not during IVM. In particular, the first 24 h after IVF was the critical period for TUDCA treatment of IVM embryos as well as the other period of preimplantation. To our knowledge, this is the first report to optimize the concentration and timing of TUDCA through cumulus-free IVM-IVF procedures to full-term development.

TUDCA relieves ER stress by acting as a chemical chaperone [17]. The ER is plays a major role in the processing and transport of proteins and lipids, as well as in calcium homeostasis. ER stress can be induced by the accumulation of misfolded proteins, which disrupts ER function and leads to ER stress-induced apoptosis. Recently, it has been reported that ER stress is induced during IVM in pigs [11]. Interestingly, ER stress occurs in cumulus–oocyte complexes and cumulus cells during IVM, but not in denuded oocytes. This is consistent with our results showing that TUDCA treatment in IVM is not effective to improve cumulus-free oocyte quality.

Treatment of embryos with TUDCA improves development of preimplantation of *in vivo* fertilized embryos in mice and pigs by attenuating apoptosis, presumably reducing ER stress.^12-14^ ER stress affects pig embryo cleavage kinetics, and the ability of embryos to develop to the blastocyst stage [14]. Although increased ER stress and genome damage are present in some cleaving embryos, relief of ER stress by TUDCA decreased genome damage to rescue the developmental competence of poorly developing embryos [11]. Our results clearly show that TUDCA treatment improves preimplantation development of embryos produced by IVM-IVF.

Through this study, we also found that a higher concentration of TUDCA (1000 μM) was more effective at supporting full-term development without overt abnormalities. Most reports tested lower concentrations, such as 50 μM, and 200 μM TUDCA [13,14], which may be due to the toxicity of TUDCA at 300 μM and above for cultured cells [18]. Our data also revealed that the toxicity of TUDCA at 1000 μM was observed during IVM but not after IVF. Therefore, TUDCA at a higher concentration could be toxic or not depending on cell types and timing. Currently, it is not clear why such a higher concentration of TUDCA is required to observe an effect in embryos. One possibility is that IVM oocytes may cause more ER stress or additional impairment to the mitochondria after IVF, as TUDCA treatment also protects against mitochondrial dysfunction [19]. Thus, the concentration of TUDCA used for treatment of IVM oocytes should be optimized differently from *in vivo* matured oocytes.

In this study, our primary objective was to optimize the concentration and timing of TUDCA treatment to improve embryo development from cumulus-free oocytes rather than to examine the mechanism underlying the improvement by TUDCA. Our results provide insights into future practical uses of TUDCA for assisted reproductive treatments. Future studies should focus on the mechanisms underlying the observed improvement when using high concentrations of TUDCA.
